# Neutrophil-to-lymphocyte ratio evolution is an independent predictor of early progression of second-line nivolumab-treated patients with advanced non-small-cell lung cancers

**DOI:** 10.1371/journal.pone.0219060

**Published:** 2019-07-17

**Authors:** Matthieu Dusselier, Elise Deluche, Nellie Delacourt, Julia Ballouhey, Thomas Egenod, Boris Melloni, Charlotte Vergnenègre, Rémi Veillon, Alain Vergnenègre

**Affiliations:** 1 Service de Pneumologie, CHU, Limoges, France; 2 Service d’Oncologie Médicale, CHU, Limoges, France; 3 CHU Bordeaux, service des maladies respiratoires, Bordeaux, France; University of South Alabama Mitchell Cancer Institute, UNITED STATES

## Abstract

**Introduction:**

Although second-line immunotherapy obtained better outcomes than chemotherapy for patients with advanced non-small–cell lung cancers (NSCLCs), it is expensive and only a minority of patients seem to benefit, based on early tumor progression post-immunotherapy. Notable host inflammation, characterized by biomarkers (e.g. neutrophil-to-lymphocyte ratio (NLR])), prolongs overall survival (OS) of surgery-, chemotherapy- and immunotherapy-treated patients. To our knowledge, no previous studies used biomarker evolution to analyze the immunotherapy impact on host inflammation. Immunotherapy mainly exerts its activity by lymphocyte reactivation.

**Methods:**

This retrospective study was conducted on patients, selected by their progression status just before their 4^th^ nivolumab injection, and treated at Bordeaux and Limoges University Hospitals. A comparative group of at least 1-year responders was also selected. Clinical parameters and hematological data just before the 1^st^ (baseline) and 4^th^ nivolumab infusions were collected to calculate the NLR change (ΔNLR) between those two infusions. The combined impact of the different known prognostic factors was also analyzed with multivariable analyses.

**Results:**

Fifty-nine patients were included. The 29 early progressors had significantly more frequent ΔNLR > 1 (*p* = 0.0007), OR 18.08 [95% CI 2.96–246.24] with progressive disease as best response to prior treatment line (*p* = 0.0014). ΔNLR < 1 prolonged OS (HR 0.001 [0.0007–0.18], *p* = 0.001); as did a partial response to prior line of systemic treatment (HR 0.14 [0.03––0.56], *p* = 0.005).

**Conclusion:**

Based on selected early progressors given second-line immunotherapy for advanced NSCLC, progression as best response to prior treatment and ΔNLR > 1 characterized the early progressors and shortened OS after starting nivolumab. This phenomenon questions nivolumab utility in patients with a major host neutrophil inflammation.

## 1. Introduction

Nivolumab was the first immunotherapy approved by the Food and Drug Administration (FDA) for advanced non-small cell lung cancer (NSCLC) second-line therapy. In France, it was given as compassionate therapy before being approved by French health authorities. Nivolumab, compared to docetaxel, has prolonged overall survival (OS) of patients with squamous [1] and non-squamous NSCLCs [2]. However, in those studies, response rates were only 42% and 19%, respectively. Those responses mean that entire population did not benefit, making it critical to identify biomarkers of patients likely to respond. Nivolumab inhibits programmed cell-death protein-1 (PD-1)–mediated signaling by blocking its ligand (PD-L1])from binding to it [3], thereby preventing reactivation of cytotoxic activity [4] and expansion of clonal T cells recognizing tumor-specific antigens [5].

However, the chronic inflammation induced by tumor development also affects the tumor’s growth, dissemination and immunoresistance [6]. Research has focused on immunological biomarkers that might identify and follow the equilibrium between pro-tumor and anti-tumor immunotherapy-caused inflammation. The neutrophil-to-lymphocyte ratio (NLR) is defined as the absolute neutrophil count (ANC) divided by the absolute lymphocyte count (ALC). Although many studies showed its interest, their results diverged. Some underlined the impact of NLR > 3.6 [7] or > 5 [8] just before the 1^st^ immunotherapy infusion (baseline) on OS and progression-free survival (PFS). Others found no NLR difference from baseline to after 6 weeks of treatment [9,10]. However, because a single ratio only catches a frozen glimpse, it is difficult to extrapolate it to the immune system’s perpetual movement. An early NLR decline, between the 1^st^ and 3^rd^ nivolumab infusions, for metastatic renal cell carcinoma patients was associated with better outcomes [11]. A study on 19 highly heterogenous NSCLC patients [12] underlined the influence of an NLR decrease on the time to treatment failure.

In this novel study, by monitoring the NLR evolution between the 1^st^ and 4^th^ nivolumab infusions, we aimed to determine whether NSCLC patients’ inflammation-biomarker evolutions impacted immunotherapy efficacy.

## 2. Methods

### 2.1 Patients and data collection

This multicenter retrospective study included 59 patients over 18 years old, receiving second- or third-line nivolumab (3 mg/kg intravenously every 2 weeks), after one or more prior chemotherapies, between June 2015 and April 2018 at Limoges and Bordeaux University Hospitals. Previous studies [1,2] assessed the first tumor response at week 9, after 4 injections. Early progressors were defined by a progression at this first evabuation, according to to the Response Evaluation Criteria In Solid Tumors guidelines for immunotherapeutics (iRECIST version 1.1) [13]. A control group of long-term responders, defined by radiologic response or stabilization under immunotherapy lasting at least 1 year, was also selected. Patients were excluded when [1] they had received first-line immunotherapy, [2] died before the 2^nd^ nivolumab infusion, [3] had a concomitant infection involving an immunodeficiency or autoimmune disorder, [4] were participating in another clinical trial or [5] were under guardian or trusteeship. Electronic medical records and pharmacy databases were screened to obtain patients’ specific information. Data collected included: demographics; smoking history; histology; endothelial growth factor-receptor (*EGFR*), anaplastic lymphoma kinase (*ALK*), transmembrane tyrosine-kinase receptor (*ROS1*) and Kirsten rat-sarcoma viral oncogene (*KRAS*) gene mutations, and PD-L1 status, when available; metastatic sites at initial diagnosis; description of previous treatments (numbers of cycles, time under treatment, best response to previous treatment[s]) number of nivolumab infusions received; response status; date of progression (or last follow-up) as determined by radiology reports; and date of death or last follow-up. Hematological and biochemistry parameters of interest (absolute leukocyte (ALC), neutrophil (ANC) and platelet (APC) counts, albumin (ALB) concentrations enabling calculation of NLR [8], ΔNLR, ΔANC, ΔALC, platelet-to-lymphocyte ratio (PLR) [7], lactate dehydrogenase (LDH), C-reactive protein (CRP) at the 1^st^ and 4^th^ infusions, the advanced lung-cancer inflammation index (ALI; (body mass index × albumin)/NLR [13] and the Lung Immune Prognostic Index (LIPI) [14].

As we compared the clinical and biological characteristics of early progressors and long responders, we studied which component had an impact on the response duration to nivolumab. Retained cut-offs were 6 Giga/L for neutrophils (upper limit of normal, ULN), NLR = 5 [7,8], and PLR = 169 and 262 [7]. An exploratory analysis evaluated the impact of ΔNLR on OS, by dividing the population according to a cut-off value of 1 chosen because the NLR standard deviation (SD) was 0.8. The median ΔANC and ΔALC were used as the dividing thresholds. OS was defined as the number of months between the 1^st^ nivolumab infusion and death or the last follow-up.

The Ethics Committee of the Limoges University Hospital approved this study (no. 285-2018-51) and informed consent was not required because of the retrospective character of the study.

### 2.2 Statistical analyses

All collected data were analyzed using Statview software (SAS Institute, Inc., Cary, NC) and R software. Quantitative results are expressed as median [range] or mean ± SD and qualitative results as *n* (%) Nominal variables were compared between groups using the chi-square or Fisher’s exact test, as appropriate. Means were compared with the non-parametric Mann–Whitney *U*-test for continuous variables. Univariate analyses identified variables associated with therapeutic response (*p* ≤ 0.2) that were then entered into multivariate logistic-regression models. The Kaplan–Meier method was applied to evaluate the OS probability. Median OS rates were compared with the non-parametric log-rank test. Multivariate Cox regression analyses of the variables achieving *p* < 0.20 in univariate analyses were used to assess nivolumab impact on OS. For all analyses, *p* < 0.05 defined significance.

## 3. Results

### 3.1 Baseline characteristics

The clinical characteristics of the 59 patients are reported in Table 1. Early progressors received a median (range) of 3 infusions during 1.3 (0.5–1.8) months vs 35 (23–47) infusion during 18 (12–33) months for the long responders. Early progressors’ median age was significantly older. The vast majority of the patients (90%) had an Eastern Cooperative Oncology Group Performance Status (ECOG PS) of 0/1 vs ≥2 for the remaining 10%. Early progressors had predominantly (69%) non-squamous NSCLCs, vs 90% for long responders (*p* = 0.01). PD-L1 testing rates were comparable for the 2 groups. Early progressors had significantly higher percentages of bone metastases (*p* = 0.01) and progressive disease (*p* = 0.001), with significantly shorter times to disease progression on previous treatment lines (*p* = 0.04).

**Table 1:** Baseline clinical characteristics of NSCLC patients receiving nivolumab as second-or-more-line therapy.

Characteristic	General population *N* = 59	Early progressors *N* = 29	Long responders *N* = 30	Univariate *p*
**Age (years), median (range)**	59.5 (30.3–87.3)	65.4 (37.8–87.3)	56.8 (30.3–77.8)	0.06
**Sex**				0.24
**Men, *n* (%)**	44 [75]	23 [79]	21 [70]	
**Women, *n* (%)**	15 [25]	5 [17]	10 [33]	
**Tobacco consumption [packs-year], mean [±SD]**	37.4 ±16.9	39.1 ±14.1	36.3 ±18.6	0.26
**Weight [kg], median [range]**	67 [43–110]	71.5 [51–110]	62 [43–95]	0.01
**Body mass index [kg/m**^**2**^**], median [range]**	23.3 [14.9–34.3]	25.3 [17.7–34]	22.2 [14.9–34.3]	0.01
**ECOG PS, *n* [%]**				0.71
**0**	28 [57]	14 [48]	14 [47]	
**1**	25 [42]	13 [45]	12 [40]	
**2**	6 [10]	2 [7]	4 [13]	
**Histology, *n* [%]**				0.01
**Non-squamous**	47 [80]	20 [69]	27 [90]	
**Squamous**	12 [20]	9 [31]	3 [10]	
**PD-L1 tested**				0.37
**No, *n* [%]**	45 [76]	23 [79]	22 [73]	
**Yes, *n* [%]**	14 [24]	5 [17]	9 [30]	
**% PD-L1 expression, median [range]**	20 [0–80]	1 [0–20]	50 [1–80]	0.16
**Metastatic sites**				
**Brain, *n* [%]**	8 [14]	4 [14]	4 [13]	0.99
**Lung, *n* [%]**	32 [54]	14 [48]	18 [60]	0.4
**Liver, *n [*%]**	14 [24]	10 [34]	4 [13]	0.06
**Adrenal gland, *n* [%]**	7 [12]	2 [7]	5 [19/17]	0.42
**Bone, *n* [%]**	9 [15]	8 [28]	1 [3]	0.01
**Pleura, *n* [%]**	6 [10]	3 [10]	3 [10]	0.9
**Prior systemic therapy, *n* [%]**				0.57
**1**	49 [83]	25 [86]	24 [80]	
**2**	9 [15]	5 [17]	4 [13]	
**≥3**	1 [2]	0	1 [3]	
**Platinum-based chemotherapy, *n* [%]**	58 [98]	27 [93]	30[100]	0.47
**No. of 1**^**st**^**-line cycles, median [range]**	5 [1–23]	4.5 [2–14]	5 [1–23]	0.02
**Days on 1**^**st**^**-line systemic therapy mean [±SD]**	167 ±163	115 ±105	214 ±192	0.04
**Best response to 1**^**st-**^**line therapy, *n* [%]**				0.001
**Partial response**	22 [37]	6 [21]	16 [53]	
**Stability**	16 [27]	7 [24]	9 [30]	
**Progression**	21 [36]	15 [52]	6 [20]	
**Nivolumab infusions, median *n* [range]**	20 [2–47]	3 [2–4]	35 [23–47]	<0.0001
**Months of nivolumab treatment, median [range]**	11 [0.5–33]	1.3 [0.5–1.8]	18 [12–33]	<0.0001

SD, standard deviation; *n*, number; ECOG-PS, Eastern Cooperative Oncology Group performance status

### 3.2 Immunological biomarkers

Biomarker results at baseline and the 4^th^ nivolumab infusions are given in Table 2. Baseline blood counts and NLR_i0_, PLR_i0_, LIPI_i0_, and ALI_i0_ were comparable for the 2 groups. Almost equal numbers of long responders (11 patients, 52.2%) and early progressors (10 patients, 47.5%) had NLR_i0_ > 5. However, at the 4^th^ infusion, early progressors were characterized by significantly higher ANC, and CRP and LDH concentrations, more frequent NLR_i4_ >5 and higher LIPI scores. In addition, ΔNLR > 1 differed significantly between the 2 groups (in univariate (*p* = 0.001) and multivariate (*p* = 0.0007) analyses). Closer examination of the 2 variables comprising the ΔNLR showed that ΔALC values differed significantly but not ΔANC.

**Table 2:** NSCLC patients’ biomarker values at the 2 times of interest.

Marker	General population *N* = 59	Early progressors *N* = 29	Long responders *N* = 30	Univariate *p*	Multivariate *p*
**Just before the 1**^**st**^ **infusion [baseline]**					
**Leu [Giga/L]**	8.1 [2.9–30.8]	8.36 [3.1–17.67]	7.97 [2.93–30.8]	0.43	
**ANC [Giga/L]**	5.4 [0.7–28]	5.9 [0.69–13.94]	4.82 [1–28]	0.22	
**ALC [Giga/L]**	1.6 [0.3–3.3]	1.71 [0.45–2.56]	1.56 [0.29–3.34]	0.92	
**APC [Giga/L]**	298 [66–617]	288 [66–617]	309 [100–500]	0.57	
**Albumin [g/L]**	36.4 [20.8–49.9]	36 [23–49]	36 [20–44]	0.63	
**CRP [mg/L]**	23.5 [1–394]	27.5 [1–394]	20.5 [1–223]	0.73	
**LDH [IU/L],**	391 [174–679]	384 [285–597]	428 [174–679]	0.64	
**NLR, *n* [%]**					
**<5**	21 [36.2]	17 [59]	20 [67]	0.5	
**>5**	37 [63]	11 [38]	10 [33]		
**PLR, *n* [%]**					
**<169**	19 [32]	9 [31]	10 [33]	0.3	
**169–262**	31 [53]	17 [59]	14 [47]		
**>262**	8 [14]	2 [3]	6 [20]		
**LIPI, *n* of patients [%]**					
**No. of data collected**	16 [27]	9 [31]	7 [23]	0.81	
**0, good**	4 [25]	3 [33]	1 [14]	0.53	
**1, intermediate**	9 [56]	5 [56]	4 [57]		
**2, poor**	3 [19]	1 [11]	2 [29]		
**ALI, *n* [%]**					
**<18**	20 [34]	9 [31]	11 [37]	0.86	
**>18**	33 [56]	17 [59]	16 [53]		
**Just before the 4**^**th**^ **infusion**					
**Leu [Giga/L], median [range]**	5 [2.7–25.1]	9.77 [4.69–25.58]	7.1 [2.7–25.1]	0.0005	
**ANC [Giga/L], median [range]**	5 [1.7–23.7]	7 [2.94–23.69]	3.88 [1.7–7]	<0.0001	
**ALC [Giga/L], median [range]**	1.7 [0.4–3.7]	1.69 [0.43–3]	1.77 [0.37–3.59]	0.29	
**APC [Giga/L], *n*, [%] median [range]**	284 [105–490]	314 [123–490]	261 [105–471]	0.19	
**Albumin [g/L], median [range]**	36.7 [14–48.1]	32.95 [14–48]	37.65 [27–44]	0.05	
**CRP [mg/L], median [range]**	22 [1–203]	46 [1–203]	13 [1–99]	0.003	
**LDH [IU/L], median [range]**	354 [170–758]	413 [196–758]	325 [170–441]	0.04	
**NLR *n*, [%]**					
**<5**	39 [66]	13 [45]	26 [87]	0.005	
**>5**	19 [32]	14 [48]	5 [17]		
**PLR *n*, [%]**					
**<169**	6 [44]	10 [34]	16 [53]	0.37	
**169–262**	21 [36]	10 [34]	11 [37]		
**>262**	11 [19]	7 [24]	4 [13]		
**LIPI *n*, [%]**					
**Data collected**	20 [33/34]	11 [38]	9 [30]		
**0, good**	7 [35]	1 [9]	6 [67]	0.01	
**1, intermediate**	9 [45]	6 [55]	3 [33]		
**2, poor**	4 [20]	4 [36]	0		
**ALI *n*, [%]**					
**<18**	18 [31]	5 [17]	13 [43]	0.07	
**>18**	34 [58]	20 [69]	14 [47]		
**Evolution**					
**ΔNLR, median [range]**	–0.18 [–17.68–+14.99]	0.65 [–13.24–+14.99]	–0.73 [–17.68–+2.91]	0.01	
**ΔNLR < 1, *n* [%]**	43 [73]	15 [52]	28 [93]	0.001	0.0007
**ΔNLR > 1, *n* [%]**	14 [24]	12 [41]	2 [7]		
**ΔANC [Giga/L], mean [±SD]**	–0.3 ± 5	1.3 ± 4.8	–1.7 ± 4.9	0.07	
**ΔALC [Giga/L] average[±SD]**	0.1 ± 0.7	–0.024 ± 0.7	0.2 ± 0.6	0.02	
**ΔPLR, median [range]**	2.27 [–310–+395]	16.44 [–222–+395]	–23.44 [–310–+145]	0.008	

Abbreviations: Leu, leukocytes; ANC, absolute neutrophil count; ALC, absolute lymphocyte count; APC, absolute platelet count; CRP, C-reactive protein; LDH, lactate dehydrogenase; NLR, neutrophil-to-lymphocytes ratio; PLR, platelet-to-lymphocyte ratio; LIPI, Lung Immune Prognostic Index; ALI, Advanced Lung-cancer Inflammation Index; SD, standard deviation; *n*, number.

### 3.3 Exploratory analyses of OS

**Fig 1:**
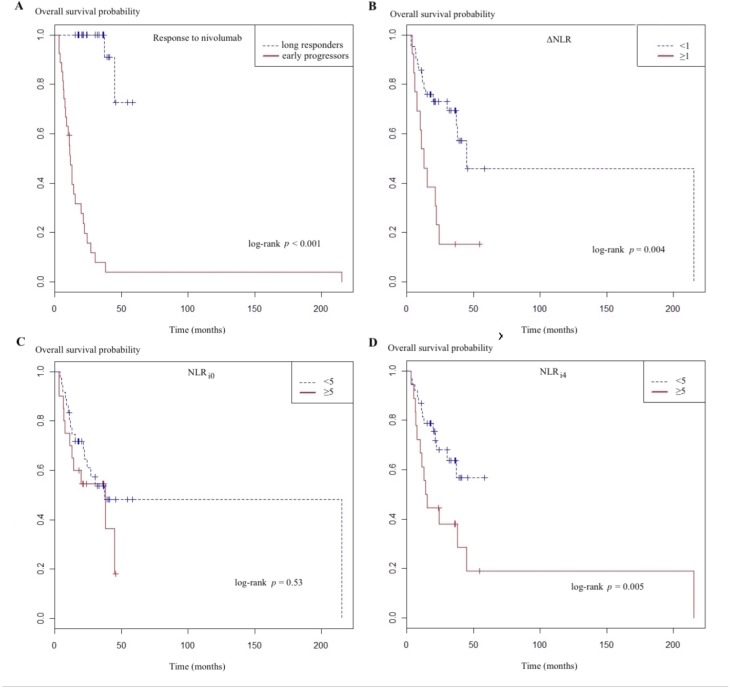
**Overall survival probability, according to the response to nivolumab (A), ΔNLR (B), NLR**_**i0**_ **(C)**, **and NLR**_**i4**_ **(D),** (A) response to Nivolumab, (B) ΔNLR (NLR change between the 1^st^ and 4^th^ nivolumab infusions), (C) NLR_i0_ (neutrophil-to-lymphocytes ratio; i0, just before the 1st nivolumab infusion), ((D) NLR_i4_ (neutrophil-to-lymphocytes ratio i4, just before 4^th^ nivolumab infusion.

Results of univariate and multivariate analyses of variables associated with OS are reported in Table 3. Among the parameters considered, univariate analyses selected only non-squamous histology as being associated with longer OS. NLR_i0_ > 5, PLR_i0_ > 262 and PLR_i4_ > 262 had no impact on OS, but NLR_i4_ > 5 did (hazard ratio (HR) 0.41 (95% CI 0.19–0.90), *p* = 0.03) (Fig 1). The multivariate model included histology, first-line–therapy characteristics (number of cycles, duration, best response) and ΔNLR. Three factors significantly impacted the OS prognosis: non-squamous histology, partial response to first-line systemic therapy, which directly reflects the number of first-line–therapy cycles, and ΔNLR > 1. At the 4^th^ infusion, ANC > 6 differed significantly but not ΔANC > –0.3. However, ΔALC >0.1 was associated with significantly prolonged OS.

**Table 3:** Univariate and multivariate analysis of overall survival.

Variable	Univariate analysis			Multivariate analysis		
	HR	95% CI	p value	HR	95% CI	p value
**Male sex**	1.83	[0.69–4.85]	0.22			
**Age: >50 years**	1.35	[0.51–3.58]	0.54	2.25	[0.5–10.04]	0.29
**Smoking history: ≤30 pack-years**	0.51	[0.16–1.56]	0.24			
**ECOG-PS: 2**	1.17	[0.40–3.41]	0.76			
**Histology: non-squamous**	0.4	[0.2–0.8]	0.01	0.19	[0.04–0.77]	0.01
**PD-L1: not tested**	1.77	[0.61–5.12]	0.29			
**Radiotherapy: yes**	1.12	[0.51–2.43]	0.78			
**No. of 1**^**st**^**-line therapy cycles**	0.88	[0.80–0.97]	0.01	0.74	[0.58–0.95]	0.01
**Time on 1**^**st**^**-line therapy**	0.996	[0.99–0.99]	0.002			
**Partial response to first-line therapy**	0.3	[0.12–0.75]	0.02	0.14	[0.03–0.56]	0.005
**Just before the 1**^**st**^ **infusion [baseline]**						
**NLR > 5**	0.69	[0.32–1.94]	0.35			
**PLR > 262**	0.68	[0.27–1.70]	0.41			
**Just before the 4**^**th**^ **infusion**						
**ANC > 6 [Giga/L]**	6.11	[2.6–14.18]	<0,001			
**ALC > 1.5 [Giga/L]**	0.99	[0.61–1.63]	0.99			
**NLR > 5**	0.41	[0.19–0.90]	0.03			
**PLR > 262**	0.87	[0.38–2]	0.74			
**Evolution**						
**ΔNLR < 1**	0.29	[0.13–0.63]	0.001	0.12	[0.03–0.46]	0.001
**ΔANC > –0.3**	1.468	[0.68–3.14]	0.32			
**ΔALC > 0.1**	0.4	[0.17–0.91]	0.03			

Abbreviations: ECOG-PS: Eastern Cooperative Oncology Group Performance Score, NLR: neutrophil-to-lymphocyte ratio, PLR: platelet-to-lymphocyte ratio, ANC: absolute neutrophil count, ALC: absolute lymphocyte count, ΔNLR: NLR_i4_ –NLR_i0_, ΔANC: ANC_i4_ –ANCi0, ΔALC: ALC_i4_ –ALC_i0_

Median OS for early progressors lasted 3.1 months but was not reached for long responders (Fig 1A). For respective subgroup analyses, for ΔNLR > 1, OS lasted 4.6 months vs 29.9 months (Fig 1B); for NLR_i0_ > 5, it lasted 7.4 months vs 29.9 months (Fig 1C); and for NLR_i4_ > 5, OS lasted 3.6 months vs not reached (Fig 1D).

## 4. Discussion

According to this novel analysis of 59 second-or-more-line nivolumab-treated NSCLC patients, NLR kinetics between the 1^st^ and 4^th^ infusions differed significantly between early progressors and long responders. Our results retained a significant increase of ΔNLR < 1 as an independent prognostic factor, regardless of its baseline level. Moreover, they also demonstrated that a partial response to the treatment line preceding nivolumab was also associated with prolonged OS in response to the latter.

Nevertheless, we recognize some limitations of this study. First, this analysis of a moderately sized progressing population was retrospective, which carries the potential for selection bias and confounders. We attempted to contain those possible weaknesses by including only patients given nivolumab (to minimize the subtle differences among the different anti-PD-1 immunotherapies). Second, the sample size is small, which can explain some results or wide confidence intervals. Third, PD-L1 status was available for only a minority of the patients included (NSCLC PD-L1 expression was not yet sought in France when these patients were treated) but the percentages were similar into the 2 groups; no further analyses could be undertaken in this study. We also adjusted our multivariable analysis to prognostic variables, because we could not control for concomitant medications that might have influenced white blood cell counts. Fourth, unlike previously reported findings [1,2], the histology-type impact on OS mainly reflects the initial selection of more non-squamous cell NSCLCs enrolled in the long responders group. Because by definition long responders lived longer, that significantly different baseline characteristic for the 2 groups unsurprisingly affected the exploratory analysis of OS.

As emphasized in a previous study published on metastatic renal cell carcinoma patients [11], a significant increase of NLR > 25% between baseline and 6 weeks after starting immunotherapy was an independent factor predictive of shorter OS. The study published on 19 NSCLC patients [12] only reported an association between an NLR increase >30% between the 1^st^ and 2^nd^ (*p* = 0.014), and 2^nd^ and 3^rd^ immunotherapy infusions (*p* < 0.001) and shorter times to treatment failures; no association was found between NLR evolution and OS or PFS. Other than the smaller number and the older age of the patients that they included, the majority had recurrent or stage-III NSCLCs and received immunotherapy later during their care (3^rd^-or-more treatment line).

The first studies to demonstrate a clear association between NLR and OS focused on NLR at baseline [7,8] and 6 weeks [9]. The former two studies retained a cut-off threshold of ≥5 but they respectively divided them into tertiles or quartiles, yielding other possible cut-off options. Those results were counterbalanced by the study highlighting the importance of NLR at 6 weeks [9], in which no associations between OS and baseline NLR were identified. Our similar population in terms of ECOG PS and previous treatment lines, our median OS of 3.6 months for NLR_i4_ > 5 was close to the 2.1 months previously reported [9].

Our selected population had many characteristics in common with the patients enrolled in phase-III trials that led to nivolumab approval for NSCLC treatment [1,2]: median age of 59.5 years compared to 62 years, a small minority of patients with ECOG PS > 2 (10% vs none) and about the same percentage of patients with ≥2 prior treatment lines (17% vs 12%).

Furthermore, the response to first-line chemotherapy before nivolumab had also been described previously for melanoma [15] and NSCLC [7,16–18]. Those responses could be caused by antigens released after tumor-cell death that would stimulate the immune T-cell response and enhance the immunotherapy mechanism of action [19–21]. We included all the previously identified clinical parameters in our multivariate analyses and they remained significant.

Previous studies on NSCLC patients treated with surgery, chemoradiotherapy or chemotherapy alone showed that a high NLR is an independent factor predictive of shorter OS and poorer PFS [22–25]. This phenomenon could be explained by an antigen-driven immune response with one or more suppressive factors [26] within the tumor microenvironment, which would lead to an immune dysfunction. The immunosuppressive tumor cells in the microenvironment, such as myeloid-derived suppressor cells or tumor-associated neutrophils [6], might be involved. Those cell types showed characteristics close to those of circulating neutrophils [27], and their influence on lymphocytes [28,29] and tumor growth [30] has been proven.

Precedent studies have underlined the positive impact of higher body mass index (BMI) >25kg/m2 on OS under chemotherapy (Carboplatin Taxol) [31] for NSCLC. A study [32] on melanoma proved similar results under immunotherapy. BMI was analyzed at a single point, without studying potential previous weight loss. In our study, BMI was collected at the diagnosis, before the first line of treatment. Since most early progressors did not respond to their first line of treatment, their performans status and weight mostly decreased, but we could not obtain a sufficient data and sample sizes to properly assess reliable statistics at the time of immunotherapy start. These results only reflects the baseline characteristics at the tumor diagnosis.

Concerning the particular NSCLC immune landscape, a recent publication [33] found a predominance of neutrophils. However, we had comparable numbers of patients in each group with NLR_i0_ > 5. Moreover, we found a significantly higher ANC_i4_, but the ΔALC > –0.3 had no impact on OS. The evolution of ΔALC > 0.1 differed statistically between our 2 study groups. Overall, a high pretreatment ANC, previously described by Bagley et al [8], and at the 4^th^ infusion in our study, was associated with shorter OS, without evolving notably over time. As stated above, ΔALC > 0.1 after starting nivolumab was associated with longer OS. That finding led us to wonder whether nivolumab would still able to overcome an immune response once dominated by neutrophils and how to determine the optimal threshold of where nivolumab could be effective: can baseline NLR adequately define the immune response and predict the response to nivolumab, or would ΔNLR better characterize each patient and be more accurately establish the cut-off?

Future prospective studies on larger populations comparing these two approaches are warranted. Because nivolumab does not seem to be effective in patients with high inflammatory (neutrophil) status, further prospective studies concerning this specific population should also be conducted to clarify the treatment algorithm. For example, comparing nivolumab versus classical chemotherapy, like paclitaxel, for progressing patients with significantly elevated NLR between the 1^st^ and 4^th^ nivolumab infusions could informative.

## 5. Conclusion

The results of this original study comparing only early progressor to long responder NSCLC patients demonstrated the importance of ΔNLR as an independent factor prognostic of OS. Early progressors were characterized by ΔNLR > 1 and progression as the best response to prior treatment line. NLR evolution was also shown to have an independent influence on OS. An increasing body of evidence seems to underline the central role of neutrophils in tumor aggressiveness and the inability of nivolumab to stop and overturn neutrophils’ pro-tumor action. Further studies on larger patient populations are needed to clarify the potential use of inflammatory biomarker evolution before and under immunotherapy as predictive and prognostic indicators of outcome.

## Supporting information

S1 File Responders data set [French version]. (XLSX)

S2 File Hyperprogressors data set [French version]. (XLSX)

## Abbreviations

i0: just before the 1^st^ infusion (baseline)
i4: just before the 4^th^ infusion
Alb: albumin (g/L]) ALC, absolute lymphocyte count (Giga/L)
ALI: Advanced Lung-cancer Inflammation Index (body mass index × albumin)/NLR
ANC: absolute neutrophil (Giga/L)
APC: absolute platelet count (Giga/L)
CRP: C-reactive protein (mg/L)
NLR: neutrophil-to-lymphocyte ratio (ANC/ALC)
dNLR: derived NLR = ANC_i0_/(ANC_i0_ –Leu_i0_)
LDH: lactate dehydrogenase (IU/L)
LEU: absolute leukocyte count (Giga/L)
LIPI: Lung Immune Prognostic Index, which accords 1 point if the derived NLR >3
LDH concentration: if above the upper limit of normal (ULN) 1 point
PLR: platelet-to-lymphocyte ratio (APC/ALC)
ΔALC: ALC_i4_ –ALC_i0_
ΔANC: ANC_i4_ –ANC_i0_
ΔNLR: NLR _i4_ –NLR _i0_
